# TNFAIP3 Maintains Intestinal Barrier Function and Supports Epithelial Cell Tight Junctions

**DOI:** 10.1371/journal.pone.0026352

**Published:** 2011-10-21

**Authors:** Lauren E. Kolodziej, James P. Lodolce, Jonathan E. Chang, Jeffrey R. Schneider, Wesley A. Grimm, Sarah J. Bartulis, Xiaorong Zhu, Jeannette S. Messer, Stephen F. Murphy, Nishith Reddy, Jerrold R. Turner, David L. Boone

**Affiliations:** 1 Department of Medicine, University of Chicago, Chicago, Illinois, United States of America; 2 Illinois Math and Science Academy, Aurora, Illinois, United States of America; 3 Department of Pathology, University of Chicago, Chicago, Illinois, United States of America; French National Centre for Scientific Research, France

## Abstract

Tight junctions between intestinal epithelial cells mediate the permeability of the intestinal barrier, and loss of intestinal barrier function mediated by TNF signaling is associated with the inflammatory pathophysiology observed in Crohn's disease and celiac disease. Thus, factors that modulate intestinal epithelial cell response to TNF may be critical for the maintenance of barrier function. TNF alpha-induced protein 3 (TNFAIP3) is a cytosolic protein that acts in a negative feedback loop to regulate cell signaling induced by Toll-like receptor ligands and TNF, suggesting that TNFAIP3 may play a role in regulating the intestinal barrier. To investigate the specific role of TNFAIP3 in intestinal barrier function we assessed barrier permeability in TNFAIP3^−/−^ mice and LPS-treated villin-TNFAIP3 transgenic mice. TNFAIP3^−/−^ mice had greater intestinal permeability compared to wild-type littermates, while villin-TNFAIP3 transgenic mice were protected from increases in permeability seen within LPS-treated wild-type littermates, indicating that barrier permeability is controlled by TNFAIP3. In cultured human intestinal epithelial cell lines, TNFAIP3 expression regulated both TNF-induced and myosin light chain kinase-regulated tight junction dynamics but did not affect myosin light chain kinase activity. Immunohistochemistry of mouse intestine revealed that TNFAIP3 expression inhibits LPS-induced loss of the tight junction protein occludin from the apical border of the intestinal epithelium. We also found that TNFAIP3 deubiquitinates polyubiquitinated occludin. These *in vivo* and *in vitro* studies support the role of TNFAIP3 in promoting intestinal epithelial barrier integrity and demonstrate its novel ability to maintain intestinal homeostasis through tight junction protein regulation.

## Introduction

One of the functions of intestinal epithelial cells (IECs), in addition to nutrient and water absorption, is to provide a dynamic, semi-permeable barrier regulated by tight junctions (TJs) between adjacent epithelial cells. This selectivity provides for the control of antigen traffic through the mucosa and facilitates interaction between bacterial flora and the mucosal immune system [1]. Inflammatory bowel disease (IBD) is characterized by an abnormal response to these antigens and a persistent inflammatory state [2]. Alterations in barrier function have been implicated in murine models of IBD [3], [4], [5], [6]. Patients with Crohn's disease, ulcerative colitis or celiac disease, and some of their first-degree healthy relatives, have increased intestinal permeability [7]–[9], suggesting that decreased barrier function may predispose or contribute to the intestinal pathology of these diseases.

The acute regulation of intestinal permeability by TNF is mediated by the activation of myosin light chain kinase (MLCK) and tight junction remodeling [10], [11]. Antigen passage across the epithelium can activate lamina propria immune cells, resulting in the secretion of TNF and an increase in MLCK activity [10]. This induces changes in epithelial cell tight junctions, including the redistribution of perijunctional actin, ZO-1, claudin, and occludin from the tight junction complex, causing an increase in paracellular flux [11]. Occludin is a tight junction transmembrane protein that both regulates and organizes the tight junction structure [1]. Tight junctions are dynamic and occludin is mobile, diffusing within the junction at the membrane [12]. Occludin ubiquitination is sufficient for the disruption of the junction (including relocalization of claudins and ZO-1) and occludin internalization results in its degradation [13]. Additionally, occludin endocytosis is essential for TNF-induced tight junction regulation [14]. Ubiquitination of membrane proteins can initiate their internalization and endocytic trafficking. Mice with constitutively active MLCK display increased barrier permeability, inflammatory cytokine production and are more susceptible to IBD [15]. Patients with Crohn's disease have increased intestinal expression and activity of MLCK, implicating this TNF-mediated pathway in the reduced barrier function that is a feature of IBD [16]. Therefore, factors that control TNF-induced changes in IEC TJs may play critical roles in barrier function and the prevention of IBD.

TNF alpha-induced protein 3 (TNFAIP3, also known as A20) is an ubiquitin-modifying enzyme that negatively regulates TNF and TLR responses [17], [18], [19]. Its expression is rapidly induced by NF-κB activation, and TNFAIP3 acts in a negative feedback loop to control its own expression, along with the production of inflammatory mediators [17], [20]. The N-terminal half of TNFAIP3 encodes a deubiquitinating (DUB) domain, whereas the C-terminal half encodes a zinc finger-containing E3 ligase domain. These two enzymatic activities work together to control the ubiquitination and subsequent degradation of cellular substrates [18]. The TNFAIP3 DUB domain can remove K63-linked polyubiquitin chains from NF-κB signaling molecules, and its E3 ligase domain can mediate the formation of K48-linked polyubiquitin chains that target the substrate to the proteasome for degradation, thereby terminating inflammatory pathways [17], [18]. DUB activity must precede subsequent E3 ligase activity for degradation to occur, but the removal of K63-linked polyubiquitin chains from a target protein does not necessarily lead to K48-linked polyubiquitination and degradation [21]. TNF induces TNFAIP3 expression in all tissues including mouse and human intestinal epithelial cells [19], [22], [23], [24].

Mice with a global deletion of TNFAIP3 are hypersensitive to LPS and TNF, develop multi-organ inflammation (including intestinal inflammation), and die prematurely [17], [19]. Mice lacking TNFAIP3 specifically in IECs do not develop spontaneous gut inflammation, but are susceptible to a dextran sodium sulfate-induced model of colitis [22]. Mucosal biopsies from Crohn's disease patients with moderate to severe disease are characterized by low TNFAIP3 expression [25]. Furthermore the *TNFAIP3* gene (located on chromosome 6q23) has been identified as a susceptibility locus in genome-wide association studies for several human autoimmune inflammatory disorders including IBD, celiac disease, psoriasis, lupus, rheumatoid arthritis and type I diabetes [26], [27], [28] [29], [30], [31], [32], [33], [34]. We have recently demonstrated an association of a nonsynonymous *TNFAIP3* polymorphism (rs5029941) with IBD risk in African-Americans [35]. This functional SNP alters the ability of TNFAIP3 to DUB TNF-receptor associated protein 2 (TRAF2). Collectively, these studies point to a role for TNFAIP3 in limiting inflammation in various diseases that is likely cell and context-dependent. Several of these TNFAIP3-associated diseases including type 1 diabetes, celiac disease and inflammatory bowel disease, are known to involve loss of intestinal barrier function [36], [37], [38]. Although TNFAIP3 is expressed in human IEC and has been identified as a protein of interest in intestinal disease, it is not known whether TNFAIP3 plays a role in barrier function, or the regulation of tight junctions by TNF. The objective of this study was therefore to determine the role of TNFAIP3 expression in IEC tight junction regulation and intestinal permeability. Our results indicate that TNFAIP3 promotes intestinal barrier function and regulates TJ dynamics, including the redistribution and ubiquitination of occludin.

## Results

### TNFAIP3 is required for intestinal barrier function

**Figure 1:**
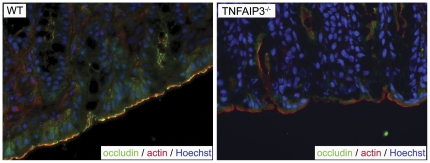
TNFAIP3 supports occludin localization at intestinal tight junctions. Immunohistochemistry for occludin (green), actin (red) and DNA (blue) is shown in intestines of untreated wild type (WT) and TNFAIP3^−/−^ mice. A lack of typical perijunctional occludin localization can be observed at the apical surface of epithelial cells in the TNFAIP3 deficient mice.

**Figure 2:**
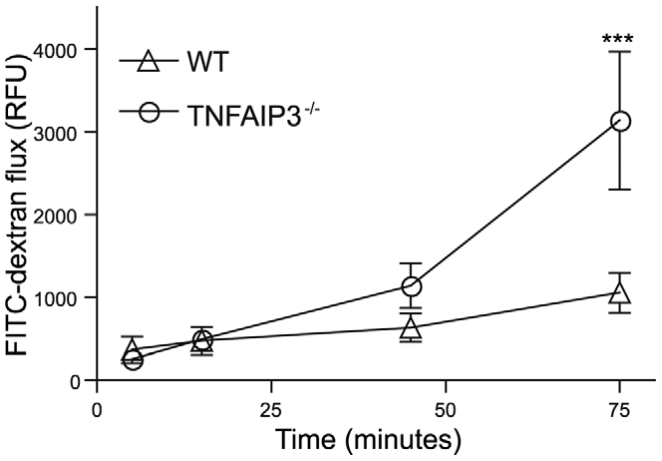
TNFAIP3 is required to maintain intestinal barrier function. Explanted intestinal loops from untreated wild type (WT; triangles) and TNFAIP3 deficient mice (TNFAIP3^−/−^; circles) were examined for loss of barrier function. The flux of FITC-dextran over time out of the loops was measured in relative fluorescent units (RFU) per cm of intestinal tissue. Increased FITC-dextran flux indicates decreased barrier function. (***p<0.001)

Factors that modulate IEC response to TNF may be critical for the maintenance of barrier function [10]. TNFAIP3 inhibits TNF-induced signaling pathways; therefore, we examined whether TNFAIP3 plays a central role in regulating IEC barrier permeability. To investigate the role of TNFAIP3 in maintaining the intestinal barrier we assessed barrier integrity in TNFAIP3^−/−^ mice. These mice spontaneously develop inflammation that is driven by TLR-dependent signals originating from endogenous microbiota [17], [19], [39]. Immunohistochemistry for the intestinal epithelial tight junction protein occludin revealed characteristic morphological features of epithelial tight junction disruption, including the loss of occludin localization from the apical surface in intestinal tissue taken from unperturbed TNFAIP3^−/−^ mice (Figure 1). Further evidence for epithelial tight junction disruption was observed when measuring flux of FITC-dextran out of explanted intestinal loops from TNFAIP3^−/−^ mice compared with that of wild-type (WT) mice. WT mice showed a minimal change in flux over time indicating an intact intestinal barrier, while TNFAIP3^−/−^ mice had a more pronounced increase in flux indicating greater intestinal permeability in these mice (Figure 2). Thus, TNFAIP3 is required for the maintenance of intestinal barrier function *in vivo*. As TNFAIP3 normally restricts inflammatory TLR signals and its expression is induced in a variety of tissue types [17], [19], [39], the increased gut permeability in TNFAIP3^−/−^ mice might reflect an indirect effect of ongoing inflammation in these mice, a direct role for TNFAIP3 in IEC TJ stability, or a combination of these two effects.

**Figure 3:**
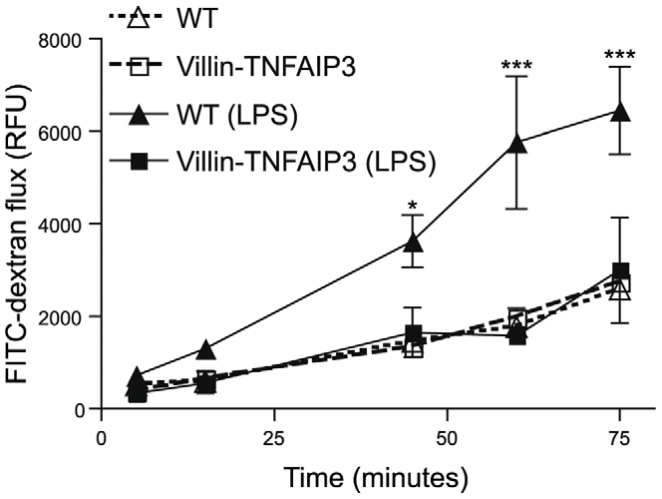
Intestinal epithelial cell-specific expression of TNFAIP3 protects against LPS-induced loss of barrier function. Explanted intestinal loops from WT (triangles) or villin-TNFAIP3 transgenic mice (squares) were assayed for barrier function beginning 45 minutes after injection of LPS (0.1 mg/mouse, ∼5 mg/kg i.p.; filled symbols) or in untreated animals (open symbols). The flux of FITC-dextran over time out of the loops was measured in relative fluorescent units (RFU) per cm of intestinal tissue. Increased FITC-dextran flux indicates decreased barrier function. (*p<0.05, ***p<0.001; WT untreated vs. WT LPS treated).

**Figure 4:**
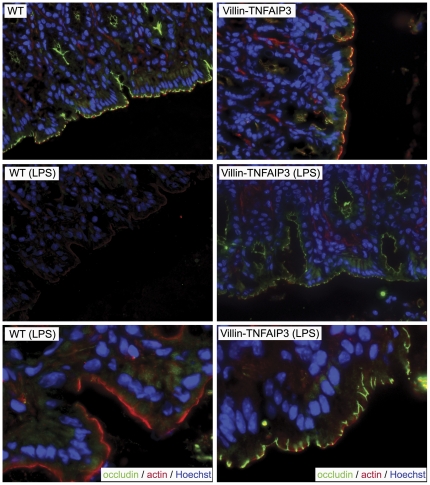
Intestinal epithelial cell-specific expression of TNFAIP3 protects against LPS-induced disruption of tight junctions. Immunohistochemistry showing occludin (green), actin (red) and DNA (blue) localization in the intestinal epithelium of untreated (upper two panels) or LPS-treated (lower four panels; 0.1 mg/mouse, ∼5 mg/kg i.p.) mice is displayed. (Upper four panels 40X objective lens; lower two panels 100X objective lens).

To assess the direct role of IEC expression of TNFAIP3 in barrier function we generated villin-TNFAIP3 mice that constitutively overexpress a TNFAIP3 transgene in the IECs of the small intestine, cecum and colon (Figures S1, S2). Villin-TNFAIP3 mice are healthy and display normal growth and reproduction up to 6 months of age. We stimulated inflammation in WT and villin-TNFAIP3 mice by injecting LPS into the peritoneal cavity 45 minutes prior to euthanasia. LPS induces a systemic inflammatory response affecting a variety of cell types including IECs, and its short-term effects are mediated largely by the induction of TNF release from innate immune cells, as well as enterocytes *in vitro* [40]–[41]. LPS induces endogenous TNF release which, when overproduced by intestinal epithelial cells, causes an IBD pathology [42]. The LPS challenge induced greater transepithelial flux of FITC-dextran across the ileum in explanted loops from WT mice compared to villin-TNFAIP3 mice, indicating that epithelial-specific overexpression of TNFAIP3 promotes resistance to inflammatory disruption of the intestinal barrier (Figure 3). Occludin is removed from apical sites of the epithelium and internalized in LPS-treated mice, and recent studies implicate occludin relocation by endocytosis in TNF-induced barrier permeability [14], [43]. Similar to these previously published reports, we observed occludin loss from the apical surface and an overall diminution of occludin intensity in IEC following administration of LPS to WT mice; however, occludin loss was markedly reduced in the intestine of LPS-treated villin-TNFAIP3 mice (Figure 4). This prevention of occludin redistribution suggests a mechanism by which TNFAIP3 affects the structure or integrity of tight junctions in IECs. Together these results demonstrate that TNFAIP3 is required for the maintenance of intestinal barrier function and that TNFAIP3 plays an important role in modulating epithelial permeability, in part by controlling occludin localization or stability in IEC.

### TNFAIP3 expression in IEC controls TNF-induced decreases in barrier function

**Figure 5:**
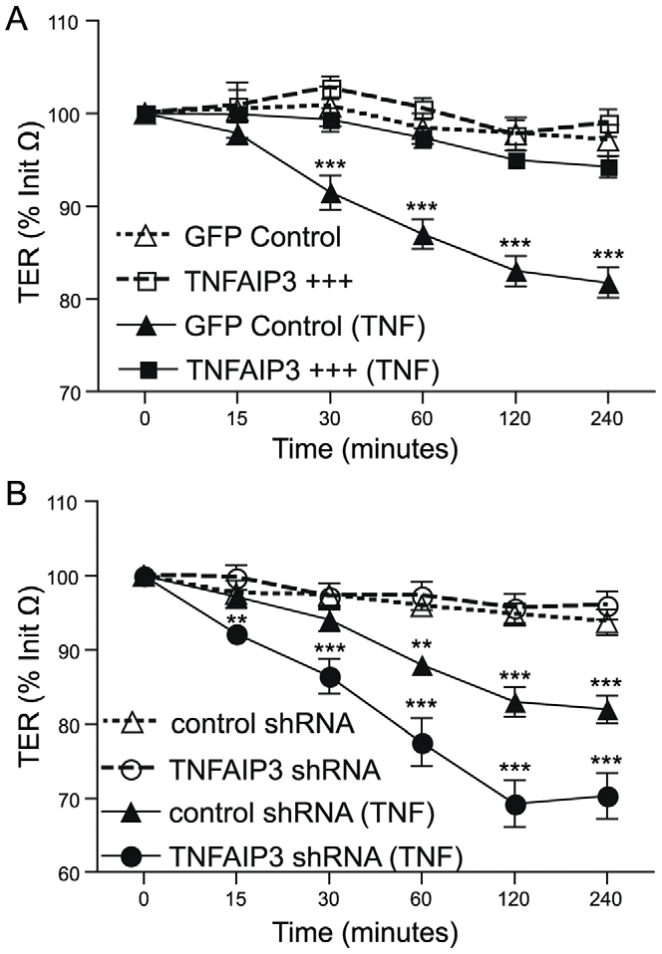
TNFAIP3 regulates TNF-induced tight junction dynamics in intestinal epithelial cells. Transepithelial electrical resistance (TER) was measured serially in cells grown in semi-permeable supports untreated (open symbols) or treated with TNF (10 ng/ml; closed symbols) for the indicated times. Values represent percentage of initial resistance measurements (% Init Ω). Pools of cells were stably transduced with (A) GFP alone (GFP control; triangles) or GFP and TNFAIP3 (TNFAIP3 +++; squares); or (B) scrambled shRNA (control shRNA; triangles) or TNFAIP3 shRNA (circles). (**p<0.01, ***p<0.001; TNF-treated GFP controls vs. untreated GFP controls (A) or TNF-treated control shRNA/TNF-treated TNFAIP3 shRNA vs. their respective untreated lines (B)).

To test whether TNFAIP3 plays a direct role in the regulation of IEC tight junction integrity, we infected the IEC line HCT116 with lentivirus containing the TNFAIP3 cDNA in order to generate epithelial cell lines that stably and constitutively overexpress TNFAIP3 (Figure S3). IECs grown in semi-permeable supports were treated with TNF in the basolateral compartment and barrier function was assessed by changes in transepithelial electrical resistance (TER) over time. TNF induced a TER drop of ∼15% (p<0.001) within 1 hour in cells expressing the control GFP vector, but this TER drop was blocked in cells overexpressing TNFAIP3 (Figure 5A). Using annexin V and propidium iodide staining we did not observe any differences in the number of dead or apoptotic cells over a similar time course, consistent with the inability of TNF alone to induce death in most cell types (Figure S4) [44]. Thus, the heterologous expression of TNFAIP3 in IECs can prevent TNF-induced decreases in barrier function in these cells.

To determine if TNFAIP3 expression is required for IEC barrier function, we infected cells with lentivirus containing TNFAIP3 shRNA constructs to generate stable pools of cells with decreased expression of TNFAIP3 (Figure S3). Cells expressing TNFAIP3 shRNA displayed a more pronounced decrease in TER, compared to cells expressing scrambled shRNA, following exposure to TNF in culture (Figure 5B). The difference in the TER change between IECs expressing scrambled shRNA compared with TNFAIP3 shRNA did not result from differences in cell death between the two cell lines (Figure S4). Thus, our studies demonstrate that TNFAIP3 expression plays a direct role in IEC to maintain barrier function during exposure to TNF *in vitro*.

### TNFAIP3 regulates barrier function downstream of MLCK

**Figure 6:**
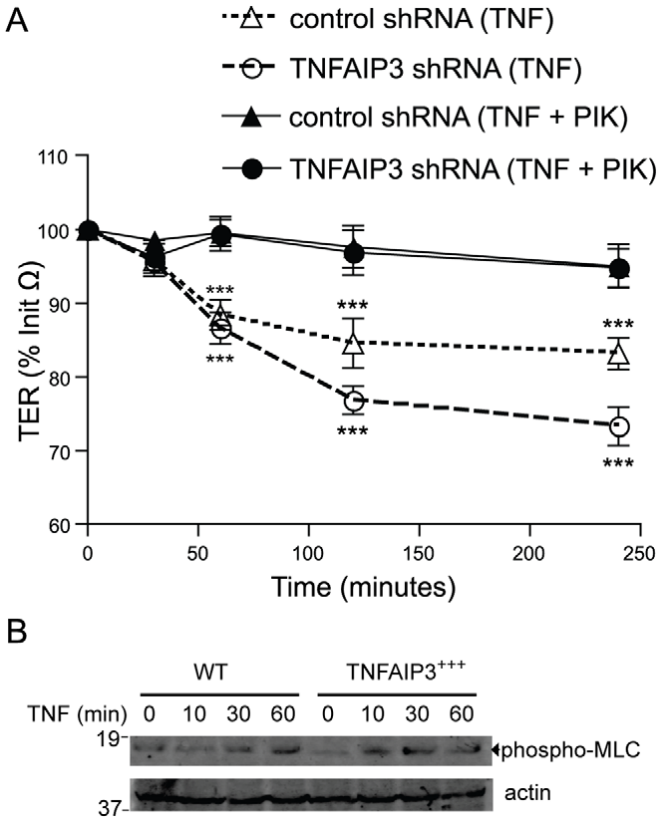
TNFAIP3 regulates an MLCK-dependent signal, but does not control MLCK activity. (A) TER measurements of stably transduced pools of cells expressing scrambled shRNA (control shRNA; triangles) or TNFAIP3 shRNA (circles) were treated with TNF alone (10 ng/ml; open symbols) or TNF plus the cell permeant MLCK inhibitor PIK (200 µM; closed symbols). Values represent percentage of initial resistance measurements (% Init Ω). (***p<0.001; TNF vs. TNF+PIK treated) (B) Immunoblot of phospho-MLC levels using whole cell lysates from GFP transduced (WT), or GFP together with TNFAIP3 transduced (TNFAIP3 +++) cells treated with TNF (10 ng/ml) for the indicated times minutes.

The control of tight junction dynamics that maintain IEC barrier function is not entirely understood, but MLCK plays a key role in the reduction of TJ integrity in response to TNF in IECs [45]. To assess whether the drop in TER associated with TNFAIP3 deficiency involved dysregulation of the MLCK signaling pathway, we treated cells with membrane-permeant inhibitor of myosin light chain kinase (PIK), a highly specific inhibitor of MLCK activity in IEC [46]. PIK did not directly inhibit TNFAIP3′s deubiquitinating activity and did not significantly change the TER of untreated cells (Figures S5, S6). As before, TNF induced a rapid decrease in TER in scrambled shRNA-expressing cells, and this decrease was more pronounced in TNFAIP3 shRNA-expressing cells. Inhibition of MLCK by PIK completely prevented the rapid decrease in TER in both scrambled and TNFAIP3 deficient cells (Figure 6A). This suggests that TNFAIP3 regulates barrier function by acting at some point in the TNF/MLCK/TJ pathway. Phosphorylation of myosin light chain (MLC) is a standard indicator of MLCK activity [10]. We therefore assessed phospho-MLC levels in TNF-treated cells to determine whether TNFAIP3 inhibits TNF-induced MLCK activation. Cells constitutively expressing TNFAIP3 did not display altered phosphorylation of MLC in response to TNF treatment (Figure 6B). These data suggest that TNFAIP3 regulates MLCK-mediated barrier function through a mechanism that does not involve inhibition of MLCK activity itself.

### TNFAIP3 deubiquitinates non-K48-linked polyubiquitinated occludin

**Figure 7:**
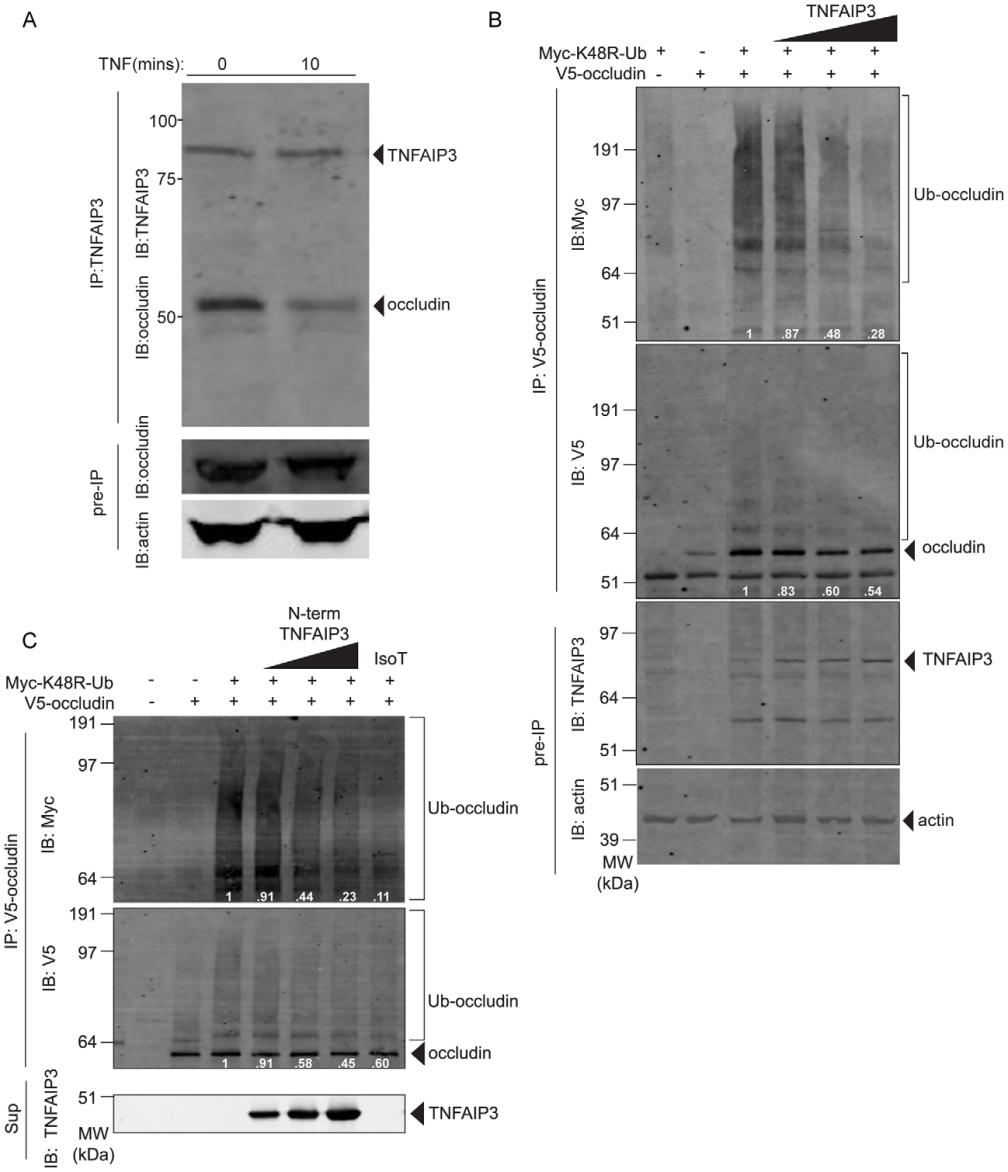
TNFAIP3 associates with and deubiquitinates occludin. (A) Immunoblot of occludin in TNFAIP3 immunoprecipitates from HCT116 cells with and without TNF stimulation. (B) Immunoblot for ubiquitin (upper panel) or occludin (center panel) from an *in vitro* DUB assay with non-K48-linked polyubiquitinated occludin and increasing amounts of recombinant N-terminal (DUB domain only, residues 1–371) TNFAIP3 (0, 5, 10, 20 µM) or isopeptidase T as a positive control (0.5 µM). Ubiquitinated occludin immunoprecipitated from transfected HEK 293T cells was mixed with enzyme for 24 hours and the reaction products were resolved by SDS-PAGE and analyzed for ubiquitination. The lower panel shows an immunoblot from the reaction supernatant (Sup) indicating the relative amount of TNFAIP3 present at the end of the assay. (C) Immunoblot from an *in vivo* DUB assay showing the ubiquitination of occludin in cells co-transfected with plasmids that express K48R-ubiquitin (0.5 µg), occludin (3 µg), and increasing amounts of full-length TNFAIP3 (0, 1, 5, 10 µg). The upper two panels are immunoblots of lysates immunoprecipitated with antibody against the V5-epitope tag (IP: V5-occludin), and the lower two panels are immunoblots of whole cell lysates (Pre-IP). In panels B and C, the inset numbers in white are the densitometry of the ubiquitin in each lane.

The ability of TNFAIP3 to regulate TJ dynamics, but not MLCK activity, suggests that TNFAIP3 might act on the TJ itself. The enzymatic activity of TNFAIP3 may directly or indirectly alter the ubiquitination of TJ proteins, such as occludin. Ubiquitination of occludin mediates its endocytosis, thus increasing permeability of the tight junction [13]. To determine whether TNFAIP3 associates with occludin in cells, we immunoprecipitated endogenous TNFAIP3 and tested for the presence of associated occludin. TNFAIP3 and occludin were associated in unstimulated cells and this association was diminished 10 minutes after stimulation with TNF. Thus, TNFAIP3 and occludin associate in cells in a manner that is regulated by TNF stimulation (Figure 7A). To determine whether TNFAIP3 can deubiquitinate occludin, we incubated recombinant N-terminal TNFAIP3 with ubiquitinated occludin *in vitro*. We found that TNFAIP3 was able to decrease the total ubiquitination of occludin (Figure 7B). In addition we transfected epitope-tagged forms of TNFAIP3, occludin, and K48R-ubiquitin into HEK 293T cells. The mutation in ubiquitin from a lysine to arginine at position 48 prevents degradative polyubiquitin chains from forming on substrates; thus, the Myc-epitope tag will mark only mono, K63-linked and other non-K48-linked polyubiquitin chains. The introduction of an increasing amount of TNFAIP3 into cells resulted in decreased ubiquitination of co-transfected occludin (Figure 7B). These results indicate that TNFAIP3 can alter the ubiquitination of occludin, and are consistent with the retention of occludin at the apical membrane in the intestines of LPS-treated villin-TNFAIP3 mice.

## Discussion

Our results demonstrate that TNFAIP3 is required to maintain barrier function in the intestine and that TNFAIP3 plays a direct role in IECs to control TNF-induced increases in IEC tight junction permeability. Furthermore, we have shown that TNFAIP3 maintains occludin localization at the apical membrane, potentially through regulation of its ubiquitination. This suggests that the role of TNFAIP3 in barrier function may result from direct action on the tight junction. The importance of TNFAIP3 in intestinal epithelial cells and cell lines has previously been demonstrated. Lineage specific deletion of TNFAIP3 sensitizes IECs to TNF-induced apoptosis *in vivo,* and TNFAIP3 regulates IEC NF-κB activation in response to TNF and TLR ligands *in vitro* [22], [23], [47], [48]. However, our findings describe for the first time a role for TNFAIP3 in the maintenance of IEC tight junctions. Thus, TNFAIP3 may play multiple roles in the regulation of IEC homeostasis including the control of NF-κB activity, apoptosis, and TJ integrity.

TNFAIP3 was initially cloned from TNF-stimulated human umbilical vein endothelial cells and was subsequently found to have limited mRNA expression in mouse and human tissues [49]. However, TNFAIP3 is a strongly NF-κB-induced gene in almost all tissues and cell types following exposure to TNF or other NF-κB-activating ligands [19], [21]. Thus, TNFAIP3 likely has essential functions in multiple cell types *in vivo*. We observed decreased barrier function in TNFAIP3^−/−^ mice, which may reflect a primary role for TNFAIP3 in IECs or a secondary effect of increased activation of immune cells in the mucosa of these mice. It is likely that TNFAIP3 plays critical roles in both epithelial cells and immune cells to control barrier function. The intestinal mucosa is continuously challenged by microbial ligands that can activate innate immune TLRs to induce inflammation. Mice adoptively transferred with TNFAIP3^−/−^ x Myd88^−/−^ hematopoietic stem cells display decreased inflammatory activation in the intestine demonstrating that the control of immune cell TLR signaling by TNFAIP3 is also required for intestinal homeostasis [39]. It has not been determined whether TNFAIP3^−/−^ mucosal immune cells cause decreased IEC barrier function, but TNFAIP3^−/−^ immune cells produce higher amounts of TNF [17]. Therefore, decreased barrier function resulting from the lack of TNFAIP3 in immune cells is one likely contributor to the disrupted IEC barrier we have observed in TNFAIP3^−/−^ mice. However, TNFAIP3 overexpression in IECs attenuates LPS-induced barrier loss *in vivo* and TNF-induced decreases in TER *in vitro*. This highlights the unique contribution of TNFAIP3 expression in IECs in directly controlling intestinal barrier function.

We found that the ubiquitination of occludin is diminished by TNFAIP3′s N-terminal DUB activity. This indicates that TNFAIP3 may limit the endocytosis of occludin caused by cytokine exposure and mediated by polyubiquitination [13]. TNFAIP3 DUB activity removes K63-linked chains from NF-κB signaling molecules such as RIP1, RIP2, TRAF2 and TRAF6; however, TNFAIP3 does not have preferential specificity for K63-linked chains [21]. The DUB domain recognizes polyubiquitinated substrates and deubiquitinates them, with higher-order ubiquitinated species more susceptible to cleavage [50]. Therefore, it is plausible that TNFAIP3-mediated ubiquitin editing contributes to the modification of tight junction proteins. We also observed association between endogenous TNFAIP3 and occludin that was rapidly diminished by TNF stimulation in WT cells. It is not known how occludin and TNFAIP3 associate with each other in cells, but both proteins are known to associate with ITCH, suggesting that TNFAIP3 and occludin may associate indirectly through ITCH. Interestingly, occludin can be polyubiquitinated by ITCH, an E3 ligase dependent on occludin phosphorylation at Ser-490 [51], [52]. ITCH-mediated occludin ubiquitination has been described in Sertoli cell TJ disruption after dibutyryl-cAMP treatment and in bovine retinal epithelial cells upon treatment with VEGF [13], [53]. Alternatively, ITCH may also facilitate deubiquitination of substrates by TNFAIP3, as ITCH^−/−^ cells display prolonged TRAF6 ubiquitination following IL-1 stimulation [52], [54]. Further studies are necessary to determine whether TNFAIP3 and ITCH work together to regulate the ubiquitination status of occludin, or act independently of one another. The kinetics of TJ opening in our studies suggest that TNFAIP3 must act rapidly to control this process. TNFAIP3 is rapidly induced by NF-κB and feeds back to deubiquitinate RIP within 30 minutes of stimulation and to stop NF-κB activation within 60 minutes of TNF treatment [18], [19]. Additionally, TNFAIP3 is phosphorylated within 15 minutes of TNF treatment, and this enhances the ability of TNFAIP3 to regulate NF-κB activation [55]. Thus TNFAIP3 associates with occludin and can deubiquitinate occludin. The rapid kinetics of TNFAIP3 regulation of other signaling pathways suggest that TNFAIP3 may control occludin ubiquitination with similar kinetics.

TNFAIP3 can inhibit both NF-κB signaling and apoptosis, but the role of TNFAIP3 *in vivo* is likely cell and context dependent, owing to the ability of NF-κB to concomitantly induce transcription of inflammatory and anti-apoptotic genes. In most cell types, stimulation with TNF will initiate an apoptotic pathway as well as expression of opposing NF-κB-driven anti-apoptotic genes. TNFAIP3 is one such anti-apoptotic gene, which in many cell types is required to prevent apoptosis, even in the setting of enhanced NF-κB activation [19]. Conversely, the loss of TNFAIP3 in B cells renders them less susceptible to apoptosis, owing to enhanced activation and production of other NF-κB-driven anti-apoptotic proteins in that cell type [56]. The role of TNFAIP3 in IECs appears best described by the former scenario, as mice with IEC-specific deletion of TNFAIP3 are sensitive to TNF-induced apoptosis and intestinal inflammation [22]. NF-κB activation is also essential for survival of intestinal epithelial cells in response to TNF, as mice lacking IKK activity display TNF-dependent IEC apoptosis and intestinal inflammation [57]. While this is likely due, at least in part, to NF-κB-induced expression of TNFAIP3, it remains to be determined whether IEC-specific expression of TNFAIP3 alone can rescue mice from the phenotype observed in the absence of IEC NF-κB activity. *In vitro*, cells typically do not undergo apoptosis when exposed to TNF alone, but require additional stressors like the concurrent addition of cycloheximide to block new protein synthesis. Consistent with this, we did not observe changes in apoptosis in cells expressing increased or diminished TNFAIP3 in our studies to assess TER following short-term treatment with TNF alone. When cells were treated with TNF and cycloheximide, for longer periods of time, the anti-apoptotic function of TNFAIP3 was evident *in vitro* (Figure S4). Our results suggest that in addition to regulating NF-κB and apoptosis, TNFAIP3 may have unique functions in IEC that promote intestinal homeostasis; namely the inhibition of TNF-induced TJ disassembly.

The overexpression of TNFAIP3 in IEC may be an attractive goal for control of intestinal inflammation in the context of human disease, as this may block both NF-κB signals and apoptosis while also preventing the loss of barrier function. The novel role of TNFAIP3 in intestinal barrier function may have consequences for inflammation in other tissues as well, as barrier function and *TNFAIP3* genetic variants have both been implicated in multiple autoimmune disorders. Although the immune system plays a major role in inflammatory disease pathogenesis, intestinal permeability may also lead to inflammatory disease. There is precedent in mouse models and humans with IBD, that gut permeability precedes the onset of inflammation [58] [3], [9] [59]. Genetic variations in *TNFAIP3* that confer loss of enzymatic function or expression may lead to IBD in part by altering TNFAIP3′s function in IEC, thereby leading to increases in intestinal permeability and disruption of intestinal homeostasis [30]. Indeed, mucosa taken from Crohn's disease patients with moderate to severe disease is characterized by low expression of TNFAIP3 [25]. Further, the lack of intestinal barrier function may impact other organs leading to, for example, diabetes or lupus, as well as other extraintestinal inflammatory disorders [60]–[62]. Thus, variations to TNFAIP3′s function as a barrier protein may have wide-ranging effects including the regulation of intestinal permeability, the innate immune system, and other organ systems.

## Materials and Methods

### Materials

Recombinant human TNF-α was from Peprotech (Rocky Hill, NJ). Myosin Light Chain Kinase inhibitor PIK was generated as described [46]. Cytokine response modifier A (CrmA) and pCMV-Myc-K48R-ubiquitin expression plasmids were generous gifts from M. Peter and C. Maki, respectively (University of Chicago, Chicago, IL). The complete murine TNFAIP3 coding sequence was PCR cloned from C57BL/6 cDNA into pCMV-Tag2 (mammalian expression vector containing an N-terminal Flag-epitope tag from Stratagene, La Jolla, CA). Human recombinant N-terminal A20 (TNFAIP3 catalytic DUB domain) and Isopeptidase T were from Boston Biochem (Cambridge, MA). Antibodies for western blotting included primary antibodies anti-V5, anti-Myc, and anti-occludin (Invitrogen, Carlsbad, CA), anti-actin and anti-HA (Santa Cruz Biotechnology, Santa Cruz, CA), anti-FLAG (Sigma-Aldrich, St. Louis, MO), anti-phospho-MLC (Cell Signaling, Danvers, MA), and anti-TNFAIP3 (generous gift from A. Ma, University of California, San Francisco, CA). Fluorescently tagged secondary antibodies included alexa-Fluor 680, 488, and 555 (Invitrogen) and IRdye 700 and 800 (Rockland Immunochemicals, Gilbertsville, PA).

### Generation of TNFAIP3-deficient and Villin-TNFAIP3 Transgenic Mice

The generation and characterization of TNFAIP3-deficient mice has been previously described [19]. Villin-TNFAIP3 transgenic mice were generated by *galK*-based recombineering (as described in [63]) of the murine BAC clone RP23-278N11, a 193,097 bp construct containing the *villin* gene and adjacent flanking genomic sequence (Figure S1). Full-length mouse TNFAIP3 cDNA containing an N-terminal FLAG-epitope tag was PCR-amplified from the pCMV-Tag2 vector described above. The final transgenic construct sequence includes 17,780 bp of promoter sequence directly upstream of the ATG start site in the *villin* gene, and measures 31,815 bp in length. The final villin-TNFAIP3 construct was digested with SalI (New England BioLabs, Ipswich, MA), and a 23,376bp linearized fragment was microinjected into C57BL/6 oocytes (University of Chicago Transgenics/ES Cell Technology Mouse Core). The transmission and expression of the transgene was confirmed in three independent lines by Southern and Northern blot analyses of mouse intestinal tissue (Figure S2). Intestinal epithelial cells from mice were isolated as described [64]. All experiments were performed with mice on a C57BL/6 background using age- and sex-matched littermate controls. All mice were housed in a specific pathogen-free facility, and the Institutional Animal Care and Use Committee at the University of Chicago approved all mouse experiments (protocol #71661 and #72089).

### Cell Culture

All experiments were performed on HCT116 cells (human colorectal carcinoma cell line; ATCC #CCL-247) and HEK 293T/17 cells (human embryonic kidney cell line; ATCC #CRL-11268). HCT116 cells were maintained in McCoy's 5A Media and HEK 293T cells were maintained in Dulbecco's Modified Eagle Medium; both supplemented with 10% (volume/volume) fetal bovine serum, and penicillin (50 units/ml)/streptomycin (50 µg/ml) (Invitrogen). Trypsin-EDTA, phosphate buffered saline (PBS), and related cell culture reagents were from Invitrogen. Cells were incubated at 37°C in 5% CO_2_ with medium changed every 2–3 days. For growth as polarized monolayers, cells were plated on collagen-coated inserts (0.4 µm pore polycarbonate, BD Biosciences, Bedford, MA) and grown on these semi-permeable supports for 21 days past confluence in McCoy's 5A Media, supplemented with 30% (volume/volume) fetal bovine serum and penicillin (50 units/ml)/streptomycin (50 µg/ml) (changed daily).

### TNFAIP3 expressing cell lines

HCT116 cell lines constitutively expressing TNFAIP3 or TNFAIP3 shRNA were developed using lentiviral transduction with vectors generously provided by Didier Trono (Ecole Polytechnique Fédérale de Lausanne, Switzerland). Lentivirus was generated in HEK 293T cells by co-transfecting three plasmids (pTAT, pMD2.G, and p89.1) containing necessary viral machinery and a fourth plasmid (pWPI or pLVTHM) containing the murine TNFAIP3 cDNA or shRNA respectively, along with an IRES-GFP. For the control cell lines, the parental pWPI vector or pLVTHM containing scrambled shRNA were used to generate virus encoding only GFP- or scrambled shRNA-expressing cells, respectively. The validated TNFAIP3 and scrambled shRNA constructs were from OriGene (Rockville, MD). Virus-containing supernatant from transfected HEK 293T cells was collected and applied to HCT116 cells. Infected, stable pools of HCT116 cells were subsequently sorted for GFP signal to greater than 95% purity by flow cytometry using the Dako Cytomation MoFlo-HTS (Carpinteria, CA).

### Intestinal Permeability Assay

To assess intestinal permeability we utilized a mouse *ex vivo* intestinal loop model previously described [65], [66]. For villin-TNFAIP3 transgenic studies, mice were injected intraperitoneally (i.p.) with 0.1 mg/mouse (∼5 mg/kg) LPS (Sigma-Aldrich) 45 minutes prior to euthanasia. Explanted intestinal segments were filled with dialyzed FITC-dextran (1 mg/mL, molecular weight 4,000, Sigma-Aldrich) in PBS, ligated at the ends with sutures, transferred into wells containing 2 mL PBS, and incubated at 37°C. To determine FITC-dextran flux across the loops, the wells were sampled at each timepoint and fluorescence was measured using BioTek Synergy2 plate reader (Winooski, VT). FITC-dextran flux was calculated as the change in fluorescence per cm of intestine over time.

### Immunohistochemistry

Immunohistochemistry was performed on frozen intestinal sections (5 µm) embedded in O.C.T. Compound (Sakura Finetek, Torrance, CA). Samples were hydrated via successive PBS washes and blocked with 5% BSA in a humidified chamber (45 minutes, 20°C) before incubation with primary antibodies (0.4 µg/ml - 2 µg/ml in 2.5% BSA, 18 h, 4°C). Samples were washed the following day with PBS, incubated with fluorescent secondary antibody (0.1 µg/ml in PBS) and Hoechst stain (2 µg/ml). Stained samples were mounted in ProLong Antifade (Invitrogen) and photographed using a fluorescence microscope (DM2500, Leica Microsystems, Buffalo Grove, IL).

### In vitro Transepithelial Electrical Resistance

TER was measured in cells grown to 21 days post confluence in semi-permeable supports (0.4 µm pore collagen-coated filters) using an EVOM epithelial voltohmmeter (World Precision Instruments, Sarasota, FL). All samples were given fresh media prior to the first TER measurement. TNF treatments (10 ng/mL) were administered basolaterally, while PIK (200 µM) treatments were given apically. TER readings were taken from two different areas of each culture well and averaged to account for variability. An empty culture well filled with the appropriate amount of media was used in each experiment to determine the approximate resistance contribution from the membrane support and media alone.

### TNFAIP3 cell death assays

TNFAIP3 overexpressing, TNFAIP3 deficient, and control lines derived from human HCT116 IECs were grown to confluency on standard 6-well tissue culture plates and treated with 0 or 10 ng/ml TNF, with or without 25 µg/ml cycloheximide (Sigma-Aldrich). Cells were harvested 3 hours post-treatment using Accutase (Innovative Cell Technologies, San Diego, CA) and incubated with 50 µg/ml propidium iodide (Invitrogen) and annexin V-biotin with streptavidin-APC (BD Pharmingen, San Diego, CA). Cell suspensions were analyzed by flow cytometry on a BD FACSCalibur (BD Biosciences) to detect cell death as indicated by PI^+^ or annexin V^+^/PI^+^ cells.

### Transfection, Immunoprecipitation and Western Blot Analysis

Transient transfections were performed as previously described [35]. For K48R-polyubiquitinated occludin immunoprecipitation (IP) studies, lysates were incubated 2 hours at 4°C with a 50% slurry of anti-V5 Ab conjugated to Protein G agarose beads (Pierce Biotechnology, Rockford, IL). Beads were recovered, washed 3 times with lysis buffer (1% Triton, 10% glycerol, 50 mM HEPES, 150 mM NaCl, 1.5 mM MgCl_2_, 1 mM EGTA, and 1% Triton X-100), and mixed with equal volume 2× Laemmli reducing buffer and boiled (100°C, 5 minutes). For phospho-MLC analysis, HCT 116 cells (GFP control and TNFAIP3 overexpressing) were treated with 10 ng/ml TNF for 30 minutes, lysed directly in Laemmli buffer, and immediately boiled as above. Proteins were resolved by SDS-PAGE (4–12% Bis-Tris NuPAGE acrylamide gel from Invitrogen) and transferred to polyvinylidene fluoride (PVDF) membranes (Millipore, Billerica, MA) using NuPAGE transfer buffer and an XCell-II blot module (Invitrogen), and then immunoblotted with the indicated primary antibodies. Fluorescent-labeled secondary antibodies were then applied and the blots were visualized using the Odyssey Imaging System (LI-COR Biosciences, Lincoln, NE).

### In vitro Deubiquitination Assays

For cell-free *in vitro* deubiquitination assays of immunoprecipitated occludin, HEK 293T cells were transfected with V5-occludin (3 µg), Myc-K48R-ubiquitin (1 µg) and CrmA (0.5 µg) for 48 hours, lysed and immunoprecipitated as described [17]. Beads were washed with lysis buffer, PBS, and then incubated 24 hours at 37°C with recombinant N-terminal TNFAIP3 (5, 10, or 20 µM) or Isopeptidase T (0.5 µM) in enzyme buffer (300 mM NaCl, 20 mM Tris, 10 mM DTT). Ubiquitination levels were assessed by SDS-PAGE and immunoblotting as described above.

### Statistical Analysis

All experiments were performed at least three times unless otherwise indicated. Results shown are the means and standard deviation of all samples taken. Statistical significance was determined using ANOVA with post-hoc Bonferroni adjustment using GraphPad Prism software (La Jolla, CA) and significance was inferred with p-values less than 0.05.

## Supporting Information

Figure S1 **Generation of the villin-TNFAIP3 transgenic construct.** Plasmid maps for generation of the villin-TNFAIP3 transgenic construct made using *galK*-based recombineering on the murine BAC clone RP23-278N11, a 193,097 bp construct containing the *villin* gene, the complete villin promoter, and adjacent genomic sequence. The parental BAC was trimmed down to contain 17,780 bp of promoter sequence upstream of the ATG start site of the *villin* gene. Full-length mouse TNFAIP3 cDNA containing an N-terminal FLAG-epitope tag was inserted in place of the villin coding sequence. The final transgenic construct measures 31,815 bp in length.
(TIF)

Figure S2 **TNFAIP3 expression in villin-TNFAIP3 mice.** (A) Northern blot analysis of TNFAIP3 mRNA expression in tissues (S, spleen; I, small intestine) from untreated or LPS-injected (0.1 mg/mouse, ∼5 mg/kg i.p.) WT or villin-TNFAIP3 transgenic mice. Endogenous and transgenic TNFAIP3 mRNA can be differentiated based on size, and the location of each band is indicated in the upper panel. Total GAPDH message is shown in lower panel as a loading control. (B) Western blot showing IEC isolated from two WT (-) and three villin-TNFAIP3 transgenic (+) mice. Lysed IEC were immunoprecipitated (IP) with an antibody against the FLAG-epitope tag and immunoblotted for FLAG to show the relative overexpression of TNFAIP3 protein in the intestine of untreated villin-TNFAIP3 transgenic mice. Whole cell lysates (Pre-IP) were blotted for actin in the lower panel.
(TIF)

Figure S3 **Generation of TNFAIP3 overexpressing and TNFAIP3 knock-down cell lines.** (A) Immunoblots of whole cell lysates from sorted HCT116 IEC lines infected with lentivirus containing GFP only (WT) or GFP together with TNFAIP3 (TNFAIP3 +++). The upper panel shows the total amount of overexpressed TNFAIP3 (center band), and the lower panel shows total actin levels. (B) HEK 293T cells were transiently co-transfected with V5-TNFAIP3 along with a scrambled negative control shRNA construct (control), or one of five different TNFAIP3 shRNA constructs (898, 899, 900, 901, 902, or 903). The upper panel shows TNFAIP3 expression in whole cell lysates immunoblotted for the V5-epitope tag. Expression of V5-TNFAIP3 is most effectively reduced using TNFAIP3 shRNA 902 (marked with an asterisk). The TNFAIP3 shRNA 902 construct was then used to make lentivirus in order to generate stable HCT116 IEC lines with knocked down expression of TNFAIP3. The lower panel shows whole cell lysates immunoblotted for actin.
(TIF)

Figure S4 **TNF alone is not sufficient to induce cell death in TNFAIP3 overexpressing or TNFAIP3 knocked-down IECs.** Cells were treated for 3 hours with 10 ng/ml TNF (+ TNF) or without (- TNF), and with 25 µg/ml cycloheximide (+ CHX) or without (- CHX). Cell death was measured using annexin V and PI. The dot plots have been gated on only GFP positive cells. The percentage of these GFP positive cells that are both annexin V positive and PI positive, or only PI positive are indicated in the quadrants and represent the cells undergoing cell death. The frequency of cell death is compared between control HCT116 cells expressing GFP alone (GFP Control) or TNFAIP3 overexpressing (TNFAIP3 +++) cell lines in (A), and between scrambled shRNA-expressing cells (Control shRNA) and TNFAIP3 shRNA-expressing cells in (B).
(TIF)

Figure S5 **The MLCK inhibitor PIK does not inhibit TNFAIP3′s deubiquitinating activity.** Recombinant N-terminal TNFAIP3 was incubated (12h, 37C) with ubiquitin chains (a mixture of di-, tri-, and tetra-ubiquitin chains) *in vitro* and reaction products were resolved by SDS-PAGE and visualized with silver staining. Deubiquitinating activity of TNFAIP3 was evident by the degradation of ubiquitin to form monomeric ubiquitin. This activity was inhibited by the cysteine protease inhibitor N-ethylmaleimide (NEM) but not by the MLCK inhibitor PIK.
(TIF)

Figure S6 **PIK does not significantly alter TER in untreated cells.** Cells expressing TNFAIP3 shRNA or control (scrambled) shRNA were treated with the MLCK inhibitor PIK and assessed for TER as described in Figure 6.
(TIF)
