# Supplementary figures and images for: TNFAIP3 Maintains Intestinal Barrier Function and Supports Epithelial Cell Tight Junctions
Source: PLoS One. 2011 Oct 21;6(10):e26352. doi: 10.1371/journal.pone.0026352 (PMC3198775; doi:10.1371/journal.pone.0026352)

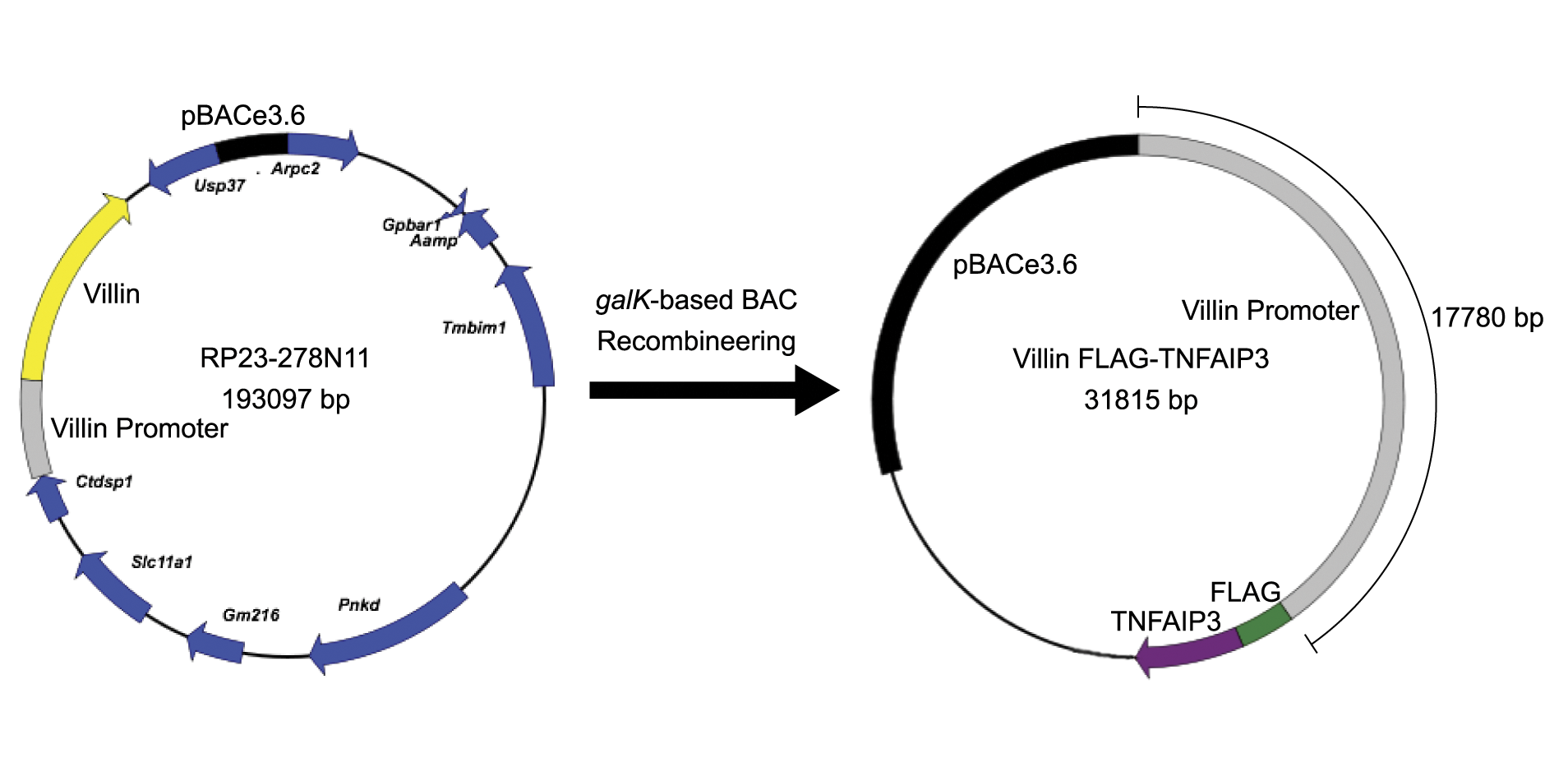

Supplement: Figure S1 — Generation of the villin-TNFAIP3 transgenic construct. Plasmid maps for generation of the villin-TNFAIP3 transgenic construct made using galK-based recombineering on the murine BAC clone RP23-278N11, a 193,097 bp construct containing the villin gene, the complete villin promoter, and adjacent genomic sequence. The parental BAC was trimmed down to contain 17,780 bp of promoter sequence upstream of the ATG start site of the villin gene. Full-length mouse TNFAIP3 cDNA containing an N-terminal FLAG-epitope tag was inserted in place of the villin coding sequence. The final transgenic construct measures 31,815 bp in length. (TIF) [file pone.0026352.s001.tif]

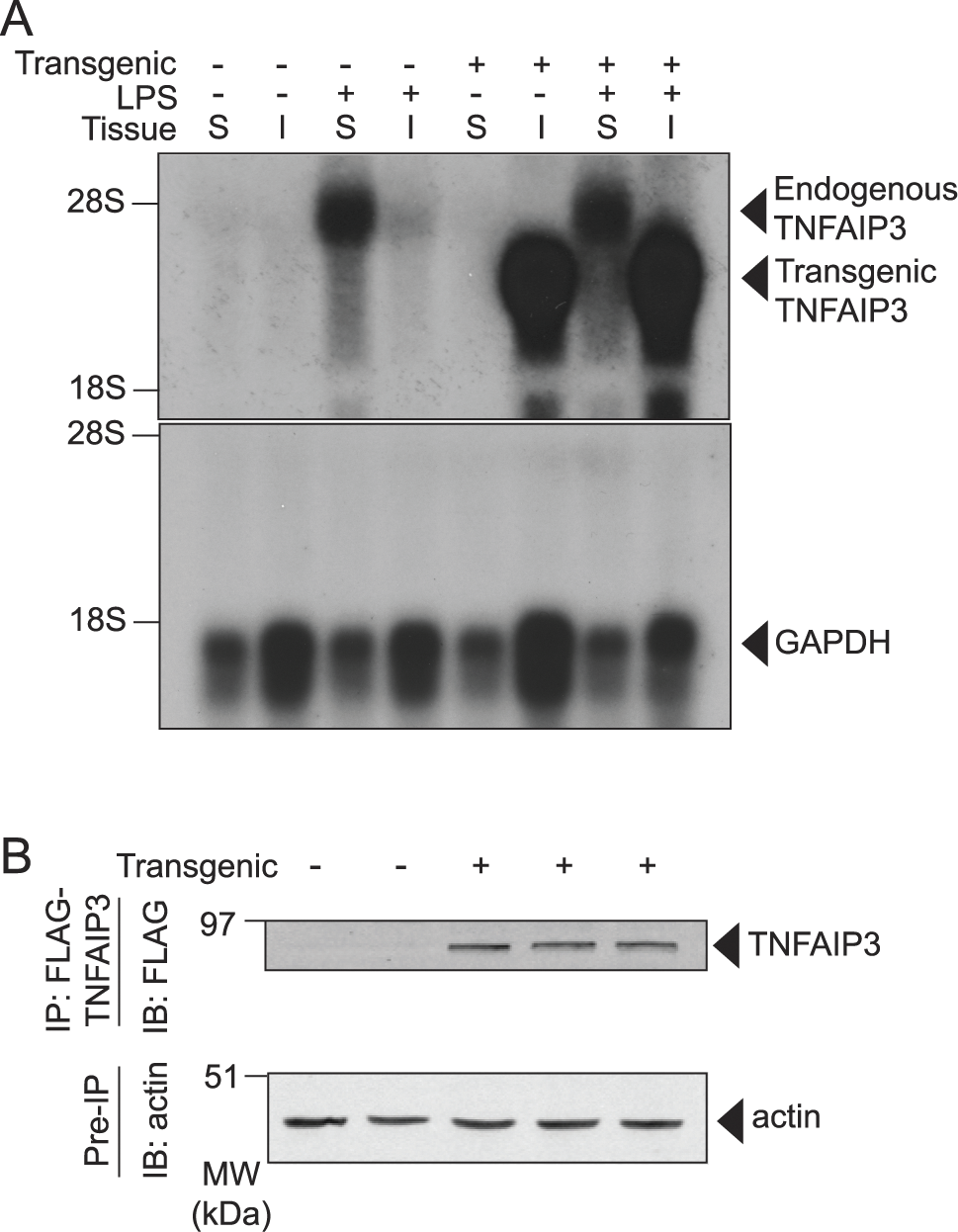

Supplement: Figure S2 — TNFAIP3 expression in villin-TNFAIP3 mice. (A) Northern blot analysis of TNFAIP3 mRNA expression in tissues (S, spleen; I, small intestine) from untreated or LPS-injected (0.1 mg/mouse, ∼5 mg/kg i.p.) WT or villin-TNFAIP3 transgenic mice. Endogenous and transgenic TNFAIP3 mRNA can be differentiated based on size, and the location of each band is indicated in the upper panel. Total GAPDH message is shown in lower panel as a loading control. (B) Western blot showing IEC isolated from two WT (-) and three villin-TNFAIP3 transgenic (+) mice. Lysed IEC were immunoprecipitated (IP) with an antibody against the FLAG-epitope tag and immunoblotted for FLAG to show the relative overexpression of TNFAIP3 protein in the intestine of untreated villin-TNFAIP3 transgenic mice. Whole cell lysates (Pre-IP) were blotted for actin in the lower panel. (TIF) [file pone.0026352.s002.tif]

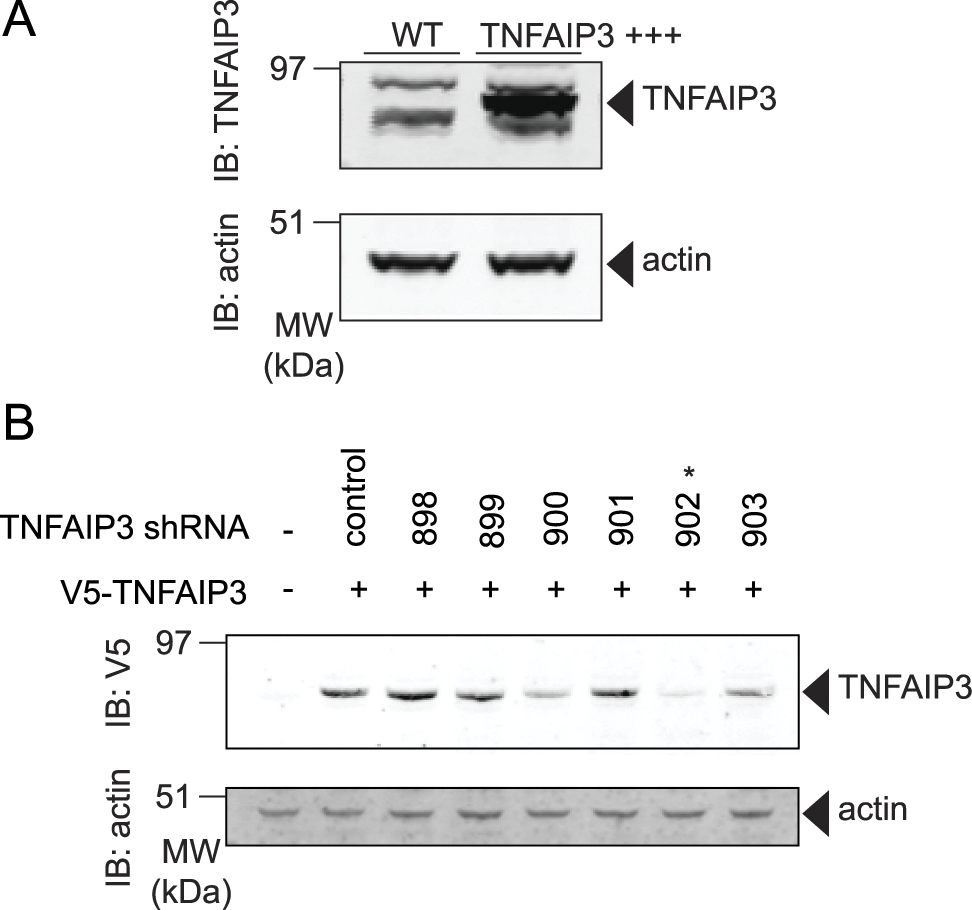

Supplement: Figure S3 — Generation of TNFAIP3 overexpressing and TNFAIP3 knock-down cell lines. (A) Immunoblots of whole cell lysates from sorted HCT116 IEC lines infected with lentivirus containing GFP only (WT) or GFP together with TNFAIP3 (TNFAIP3 +++). The upper panel shows the total amount of overexpressed TNFAIP3 (center band), and the lower panel shows total actin levels. (B) HEK 293T cells were transiently co-transfected with V5-TNFAIP3 along with a scrambled negative control shRNA construct (control), or one of five different TNFAIP3 shRNA constructs (898, 899, 900, 901, 902, or 903). The upper panel shows TNFAIP3 expression in whole cell lysates immunoblotted for the V5-epitope tag. Expression of V5-TNFAIP3 is most effectively reduced using TNFAIP3 shRNA 902 (marked with an asterisk). The TNFAIP3 shRNA 902 construct was then used to make lentivirus in order to generate stable HCT116 IEC lines with knocked down expression of TNFAIP3. The lower panel shows whole cell lysates immunoblotted for actin. (TIF) [file pone.0026352.s003.tif]

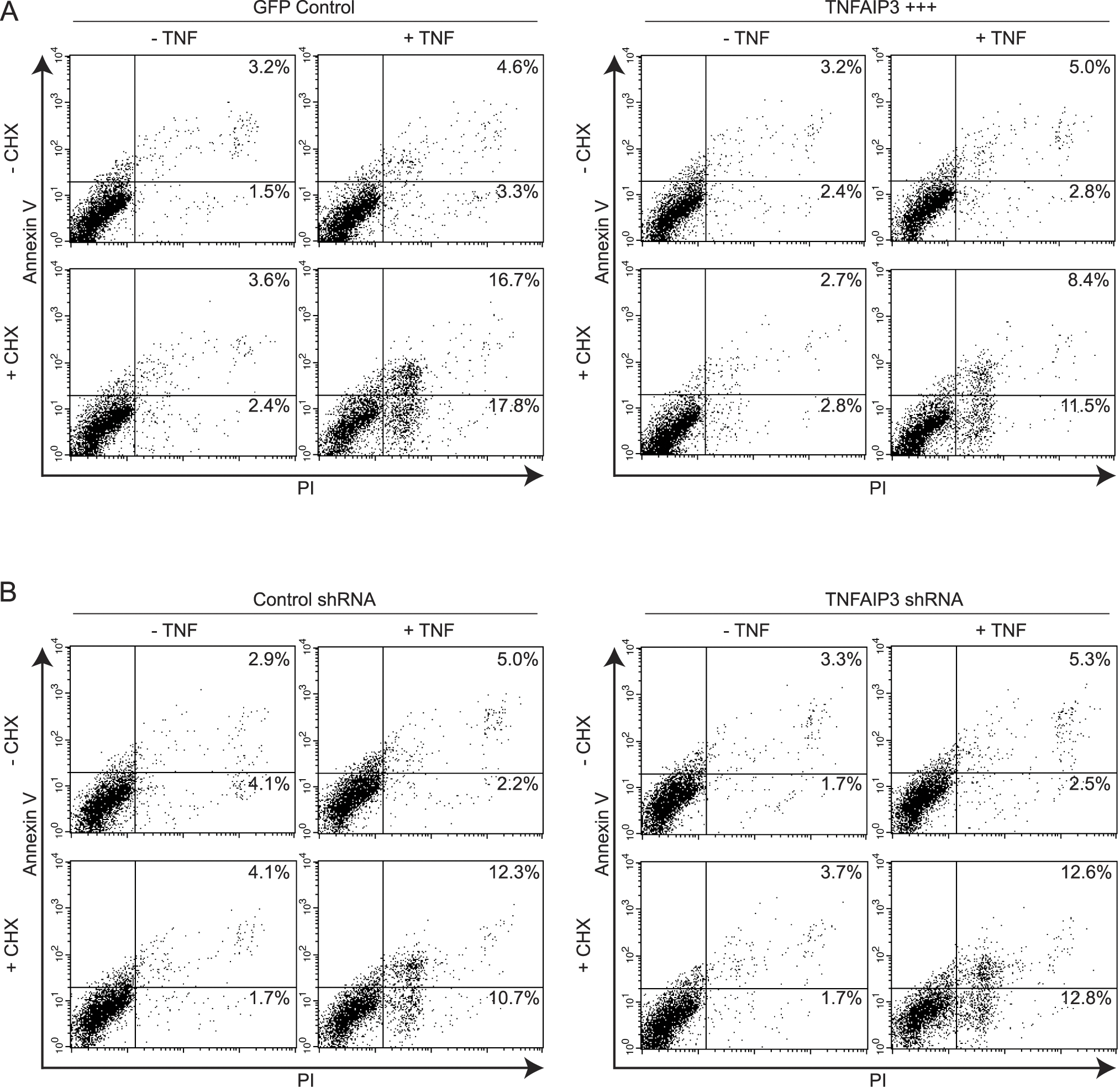

Supplement: Figure S4 — TNF alone is not sufficient to induce cell death in TNFAIP3 overexpressing or TNFAIP3 knocked-down IECs. Cells were treated for 3 hours with 10 ng/ml TNF (+ TNF) or without (- TNF), and with 25 µg/ml cycloheximide (+ CHX) or without (- CHX). Cell death was measured using annexin V and PI. The dot plots have been gated on only GFP positive cells. The percentage of these GFP positive cells that are both annexin V positive and PI positive, or only PI positive are indicated in the quadrants and represent the cells undergoing cell death. The frequency of cell death is compared between control HCT116 cells expressing GFP alone (GFP Control) or TNFAIP3 overexpressing (TNFAIP3 +++) cell lines in (A), and between scrambled shRNA-expressing cells (Control shRNA) and TNFAIP3 shRNA-expressing cells in (B). (TIF) [file pone.0026352.s004.tif]

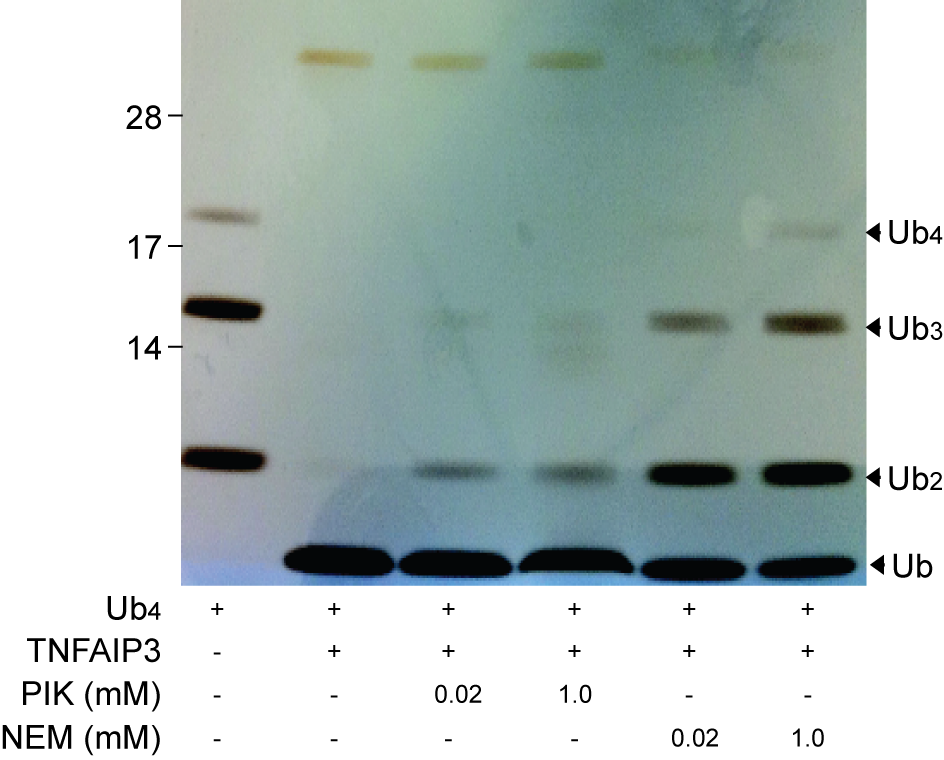

Supplement: Figure S5 — The MLCK inhibitor PIK does not inhibit TNFAIP3′s deubiquitinating activity. Recombinant N-terminal TNFAIP3 was incubated (12h, 37C) with ubiquitin chains (a mixture of di-, tri-, and tetra-ubiquitin chains) in vitro and reaction products were resolved by SDS-PAGE and visualized with silver staining. Deubiquitinating activity of TNFAIP3 was evident by the degradation of ubiquitin to form monomeric ubiquitin. This activity was inhibited by the cysteine protease inhibitor N-ethylmaleimide (NEM) but not by the MLCK inhibitor PIK. (TIF) [file pone.0026352.s005.tif]

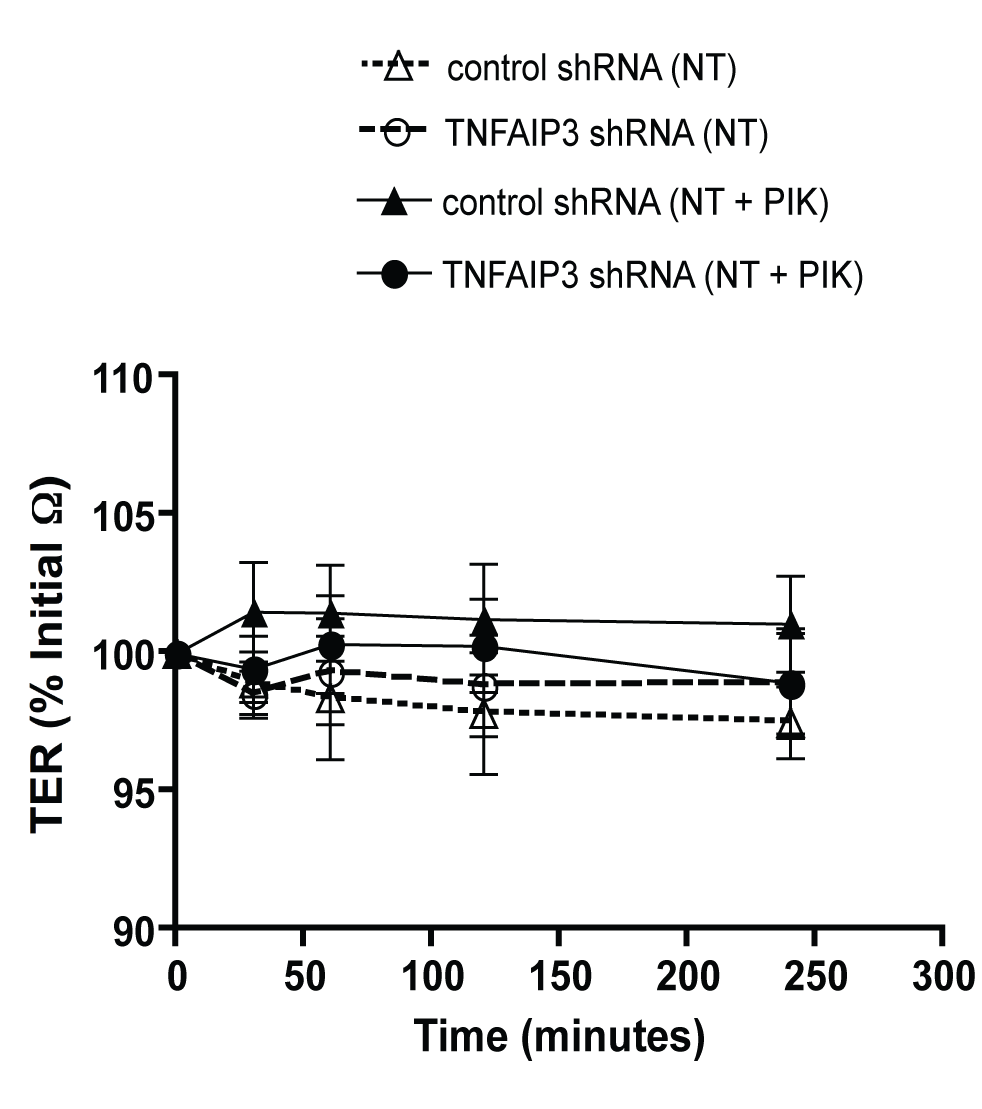

Supplement: Figure S6 — PIK does not significantly alter TER in untreated cells. Cells expressing TNFAIP3 shRNA or control (scrambled) shRNA were treated with the MLCK inhibitor PIK and assessed for TER as described in Figure 6. (TIF) [file pone.0026352.s006.tif]
